# The Impact of Intervention Design on User Engagement in Digital Therapeutics Research: Factorial Experiment With a Mixed Methods Study

**DOI:** 10.2196/51225

**Published:** 2024-02-09

**Authors:** Hyerim Lee, Eung Ho Choi, Jung U Shin, Tae-Gyun Kim, Jooyoung Oh, Bokyoung Shin, Jung Yeon Sim, Jaeyong Shin, Meelim Kim

**Affiliations:** 1 Department of Psychology College of Liberal Arts Yonsei University Seoul Republic of Korea; 2 Department of Dermatology Yonsei University Wonju College of Medicine Wonju Republic of Korea; 3 Department of Dermatology CHA Bundang Medical Center CHA University Seongnam Republic of Korea; 4 Department of Dermatology Severance Hospital, Cutaneous Biology Research Institute Yonsei University College of Medicine Seoul Republic of Korea; 5 Department of Psychiatry Gangnam Severance Hospital Yonsei University College of Medicine Seoul Republic of Korea; 6 Institute of Behavioral Sciences in Medicine Yonsei University College of Medicine Seoul Republic of Korea; 7 Department of Integrative Medicine Yonsei University College of Medicine Seoul Republic of Korea; 8 Department of Medical Device Engineering and Management Yonsei University Graduate School Seoul Republic of Korea; 9 Department of Preventive Medicine Yonsei University College of Medicine Seoul Republic of Korea; 10 Herbert Wertheim School of Public Health and Human Longevity Science University of California San Diego San Diego, CA United States; 11 The Design Lab University of California San Diego San Diego, CA United States; 12 Center for Wireless & Population Health Systems Calit2’s Qualcomm Institute University of California San Diego San Diego, CA United States

**Keywords:** atopic, dermatitis, experimental design, mobile health, patient engagement, research methodology

## Abstract

**Background:**

User engagement is crucial for digital therapeutics (DTx) effectiveness; due to variations in the conceptualization of engagement and intervention design, assessment and retention of engagement remain challenging.

**Objective:**

We investigated the influence of the perceived acceptability of experimental intervention components and satisfaction with core intervention components in DTx on user engagement, while also identifying potential barriers and facilitators to user engagement.

**Methods:**

We conducted a mixed methods study with a 2 × 2 factorial design, involving 12 outpatients with atopic dermatitis. Participants were randomized into 4 experimental groups based on push notification (“basic” or “advanced”) and human coach (“on” or “off”) experimental intervention components. All participants engaged in self-monitoring and learning courses as core intervention components within an app-based intervention over 8 weeks. Data were collected through in-app behavioral data, physician- and self-reported questionnaires, and semistructured interviews assessed at baseline, 4 weeks, and 8 weeks. Descriptive statistics and thematic analysis were used to evaluate user engagement, perceived acceptability of experimental intervention components (ie, push notification and human coach), satisfaction with core intervention components (ie, self-monitoring and learning courses), and intervention effectiveness through clinical outcomes.

**Results:**

The primary outcome indicated that group 4, provided with “advanced-level push notifications” and a “human coach,” showed higher completion rates for self-monitoring forms and learning courses compared to the predetermined threshold of clinical significance. Qualitative data analysis revealed three key themes: (1) perceived acceptability of the experimental intervention components, (2) satisfaction with the core intervention components, and (3) suggestions for improvement in the overall intervention program. Regarding clinical outcomes, the Perceived Stress Scale and Dermatology Life Quality Index scores presented the highest improvement in group 4.

**Conclusions:**

These findings will help refine the intervention and inform the design of a subsequent randomized trial to test its effectiveness. Furthermore, this design may serve as a model for broadly examining and optimizing overall engagement in DTx and for future investigation into the complex relationship between engagement and clinical outcomes.

**Trial Registration:**

Clinical Research Information Service KCT0007675; http://tinyurl.com/2m8rjrmv

## Introduction

### Digital Therapeutics in General

With the rapid advancement of digital technology, digital therapeutics (DTx) have emerged as a promising approach to either enhance the value of conventional health care delivery systems or have the potential to substantially substitute the existing system [1]. DTx refers to “an evidence-based intervention that is driven by high-quality software programs to prevent, manage, or treat a disease or disorder” [2]. Using technology and data analytics, DTx holds numerous benefits in health care: (1) it can encompass a wide range of physical and mental health conditions (mostly chronic) like diabetes, oncology treatment management, insomnia, attention-deficit/hyperactivity disorder (ADHD), and substance use disorder [3]; (2) it can provide personalized care with data-driven treatment options [4]; and (3) it can reduce health care costs [5]. Given these significant potential benefits, it is crucial to understand how the efficacy of DTx in therapy can be improved. To achieve such improvement, diverse and comprehensive research regarding the DTx development process should be conducted to successfully implement and optimize these promising interventions.

### User Engagement Issues in DTx

It is widely acknowledged that user engagement is important for improving the effectiveness of DTx [6]. Engagement in DTx can be defined as “the extent (eg, amount, frequency, duration, and depth) of use and subjective experience characterized by attention, interest, and affect” [7,8]. Although user engagement significantly impacts the effectiveness of DTx, assessing and retaining it is challenging. The possible reasons for this may include (1) a lack of shared awareness regarding the useful perception of engagement, (2) engagement in DTx is not a stationary but a dynamic process, and (3) it is a multifaceted construct, capturing the user’s behavior, cognitive, and emotional states. Several systematic reviews have investigated DTx intervention components (eg, self-monitoring, reminders, and rewards) that are linked with higher engagement [9,10]. However, the findings of these studies do not provide conclusive evidence about the intervention components that help patients become more engaged with the DTx. This occurs due to substantial variation in the definition of engagement and intervention design in DTx. Thus, an in-depth analysis of the intervention components and a concrete definition of user engagement should be established, particularly during the design phase of DTx.

### Methods for Evaluating Intervention Components in Digital Intervention

For systematically evaluating how intervention design influences user engagement, the optimization methods from the multiphase optimization strategy (MOST) can be used with a couple of representative intervention components from a wide range of possible options. MOST allows for efficient testing through a randomized experiment, including a factorial experiment, which allows for the simultaneous examination of different intervention design factors [11]. Many recent studies, however, used only traditional randomized controlled trials (RCTs) as the primary study design to test the efficacy of the intervention as a package [12-15] and to examine the relationships between engagement level and clinical outcomes through post hoc analysis [6,16,17]. Using only RCTs as an evaluation design may pose some challenges to the effective evaluation of DTx, as they are considered complex, context-dependent, and individually tailored interventions that purport to maximize its effectiveness [18,19]. Thus, additional evaluation methods for DTx, such as adaptive study designs (eg, sequential multiple assignment randomized trial and factorial trial from MOST), must be considered to provide robust evidence during its design and development phases.

### Aims of This Study

**Figure 1:**
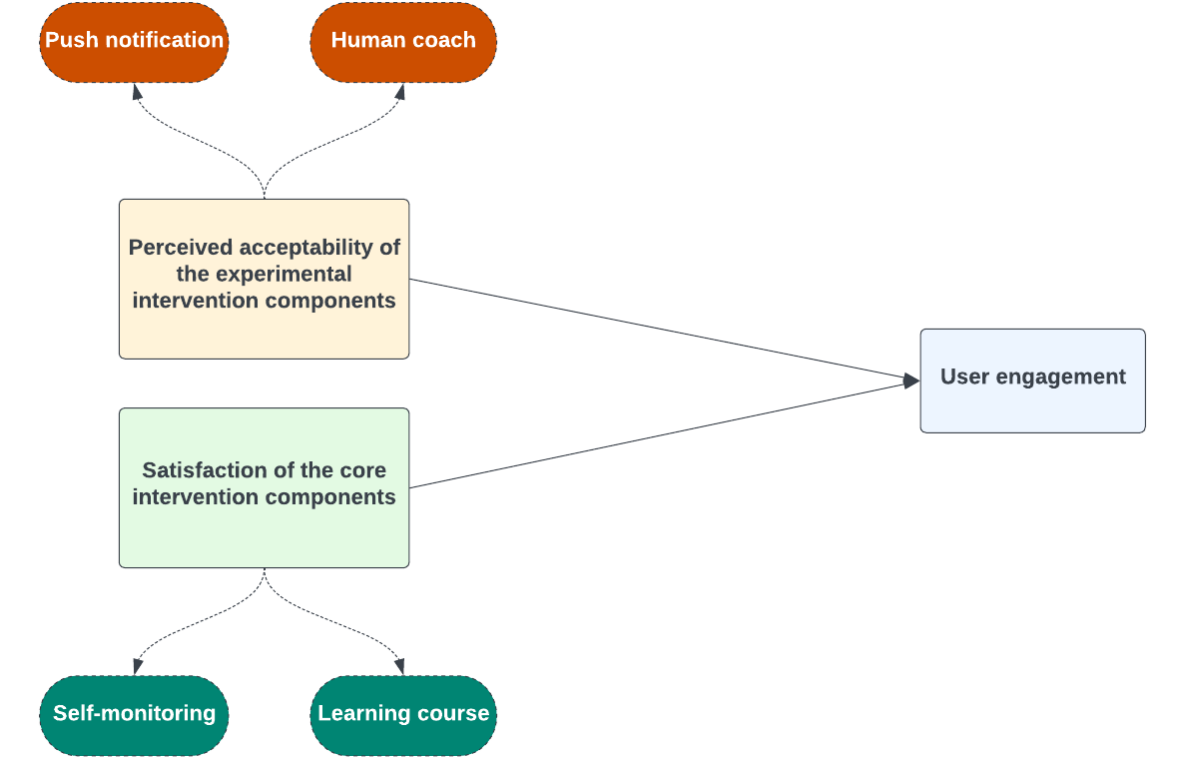
Overview of the impact of intervention designs on user engagement in “Atomind".

Here, we aimed to examine the impact of the perceived acceptability of the experimental intervention components (ie, push notification and human coach) and satisfaction with the core intervention components (ie, self-monitoring and learning courses) in DTx on user engagement (Figure 1). We used “Atomind,” a DTx for patients with atopic dermatitis (AD), developed for clinical trial purposes, with a primary focus on optimization as a refinement process before validating its effectiveness through larger RCTs. This was a proof-of-concept study with an experimental 2 × 2 factorial design, using both quantitative (eg, in-app behavioral data) and qualitative (eg, semistructured interviews) assessment methods. We hypothesized that those who received the advanced level of each experimental intervention component would pass the threshold of clinical significance of user engagement metrics in DTx. Moreover, the qualitative analysis of satisfaction with the core intervention component would identify the potential barriers and facilitators to user engagement. This study could also inform how to optimize and evaluate other DTx in this field.

## Methods

### Study Design

**Figure 2:**
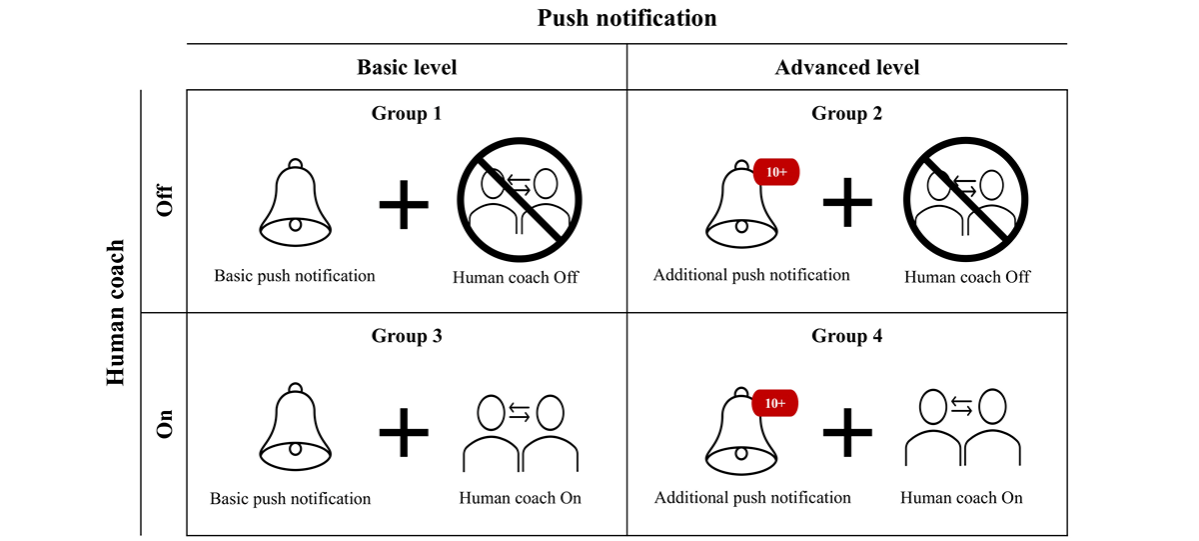
A 2 × 2 factorial design exploring the perceived acceptability of experimental intervention components in this digital therapeutics (DTx) study, featuring different combinations of basic- versus advanced-level push notification and “on” versus “off” human coach.

This full factorial experiment had 2 experimental intervention components (Figure 2), each of which was implemented at 2 different levels: push notification (“basic” or “advanced”) and human coach (“on” or “off”). Participants were randomly allocated to 1 of the 4 experimental groups in the 2 × 2 full factorial design. All participants engaged in self-monitoring and learning courses as core intervention components during the 8-week intervention period. We applied a mixed methods approach by collecting quantitative (eg, surveys) and qualitative (eg, semistructured interviews) data to examine the perceived acceptability of experimental intervention components, satisfaction with the core intervention components, and suggestions for improvement in the overall intervention program. We conducted the interviews after 8 weeks of treatment.

### Experimental Intervention Components

#### Push Notification

Participants randomized to “basic-level push notification” received basic push notifications that encouraged users to log in and complete tasks at time points chosen by users. Participants randomized to “advanced-level push notification” received not only basic push notifications but also additional push notifications when they did not complete in-app self-monitoring forms, weekly classes, or missions after receiving the basic push notifications. Additional push notifications contained emotionally supportive phrases (eg, “It’s a bit annoying, right? But don’t forget that sustained use of the app can help reduce your symptoms.” And, “Malang is waiting for <username>! Haven’t you finished the class yet? Don’t give up and let’s start!”)*.* Push notifications are classified into 4 different categories: self-monitoring, learning course, mission, and personalized feedback report. An overview of the 2 groups’ push notifications is presented in Multimedia Appendix 1.

#### Human Coach

Participants with this experimental intervention component turned “on” received tailored guidance and assistance from a human coach. The coach sent weekly motivational messages to maintain participants’ engagement through a different digital application called KakaoTalk, which is the most popular instant messaging app in South Korea, with 94% of the entire Korean population as users. The coach spent a total of 6 hours a week—2 hours a day over 3 days—to manage the participants. The coach kept the participants motivated, held them responsible, provided feedback, and monitored their progress to keep them on track. Participants could address difficulties or questions they encountered with the app through 2-way communication. Besides the app’s information, participants could also ask questions about skin health and mental well-being and receive answers from the coach. Conversely, participants with this experimental intervention component turned “off,” received nothing, and conducted self-care.

### Participants

All participants were outpatients who met the eligibility criteria, including individuals who (1) were aged 19 years or older and had mild to severe AD, (2) were able to understand verbal and written Korean, and (3) had their own smartphone. Participants who met the eligible criteria were assigned randomly to 4 experimental groups in a 1:1:1:1 ratio using program IDs generated within the Atomind app.

### Intervention

Atomind is an app-based intervention program that helps individuals manage skin conditions and AD symptoms. It was developed by Huray Inc, South Korea (Multimedia Appendix 2). The app’s content is based on cognitive behavioral theory (CBT) and a mindfulness approach to support healthy behavioral habits and regulate negative emotions. The app prompts users to complete in-app self-monitoring forms on a daily, weekly, and monthly basis, focusing on motivation, skin condition, behavioral change, and mental health. Weekly videos demonstrate educational information that can help relieve AD symptoms and CBT strategies for regulating negative thoughts and emotions. After watching the video, users were asked to demonstrate their understanding by passing a postquiz. The overall topic of the weekly video is listed in Multimedia Appendix 3. Moreover, missions are provided to help users apply their newly acquired skills in real life. Users can access personalized graphic feedback based on their self-monitoring.

### Outcomes

Outcome measures were collected by using (1) in-app behavioral data, (2) physician- or self-reported questionnaires, and (3) semistructured interviews. At baseline, participants were asked to complete a demographic questionnaire pertaining to their age, gender, educational level, and health-related measures (medical and family health history, health literacy, etc).

The primary outcome was the user engagement of the intervention, measured by in-app behavioral data on core intervention components, including percentages of self-monitoring forms and learning courses completed. We collected qualitative data on the perceived acceptability of experimental intervention components (ie, push notification and human coach), satisfaction with the core intervention components (ie, self-monitoring and learning courses), and suggestions on any improvement for the overall intervention program through semistructured interviews. The interviews were conducted over the telephone by 2 research team members after 8 weeks of intervention. A semistructured interview guide (Multimedia Appendix 4) was used to guide the interviews, lasting 15-20 minutes for each.

Furthermore, other clinical outcome measures were assessed at baseline, 4 weeks, and 8 weeks of intervention. Designated dermatologists assessed the severity of AD using the eczema area and severity index (EASI), including the severity of 4 signs (erythema, edema or papulation, excoriation, and lichenification; range 0-72) [20]. Atopic eczema severity reported by patients was measured with the patient-oriented eczema measure (POEM; range 0-28), a 7-item questionnaire for monitoring the care of patients with atopic eczema [21]. Insomnia severity was measured with the insomnia severity index (ISI; range 0-28), a 7-item questionnaire assessing perceived insomnia severity using a Likert-type scale [22]. Perceived stress level was measured with the perceived stress scale (PSS; range 0-40), a 10-item questionnaire assessing psychological stress [23]. Quality of life was measured by the dermatology life quality index (DLQI; range 0-30), a 10-item questionnaire assessing how much the patients’ skin problems have affected their lives over the past week [24], and fear of negative evaluation was measured using the brief fear of negative evaluation (BFNE; range 12-60) scale, which is a 12-item questionnaire assessing the degree of anxiety about perceived negative evaluation [25]. The assessment methods and assessment period for each measurement are shown in Multimedia Appendix 5.

### Statistical Analysis

Descriptive statistics were used to analyze quantitative data, including in-app behavioral data and clinical outcomes. We initially recruited and enrolled 12 participants, with 3 individuals for each group; however, of the initial 12 individuals, 3 participants (1 in group 2, 1 in group 3, and 1 in group 4) were excluded from the analysis due to medication changes during the intervention period.

We set the threshold of clinical significance (TCS) for user engagement, considering the period of each assessment. Previous research with larger sample sizes has shown that individuals with high efficacy typically maintain an engagement rate between 50% and 80% [26,27]. However, given the smaller sample size in this proof-of-concept study, a more stringent approach has been applied in setting the TCS for user engagement. For self-monitoring, the TCS is determined if the average completion rate of self-monitoring forms is ≥90%. For learning courses, the TCS is set if the average completion rate of learning courses is ≥80% throughout the intervention period.

The perceived acceptability of each experimental intervention component and the satisfaction of each core intervention component were also examined by semistructured interviews. Qualitative data were analyzed using thematic analysis. The verbatim transcriptions of the interviews were used to extract the responses, which were categorized into items focusing on the perceived acceptability of the experimental intervention components, satisfaction of the core intervention components, and suggestions on any improvement for the overall intervention program.

To measure the interventions’ effectiveness, we assessed the changes in the average of clinical outcomes (eg, EASI, POEM, ISI, PSS, DLQI, and BFNE) before and after the intervention in the 4 groups and the 2 different levels of each experimental intervention component.

### Ethical Considerations

All study activities were conducted in adherence to ethical standards and received approval from the institutional review boards of the organizing sites, including Severance Hospital (4-2022-0922), Wonju Severance Christian Hospital (CR322035), and Bundang Cha Hospital (CHAMC 2022-05-005-001). The trial was registered on the Clinical Research Information Service (KCT0007675). Participants provided voluntary, written, and informed consent after a thorough explanation of the clinical trial. Privacy measures included data anonymization and secure storage. Participants received US $80 in compensation for their contribution, a detail communicated during the informed consent process.

## Results

### Sample Characteristics

**Figure 3:**
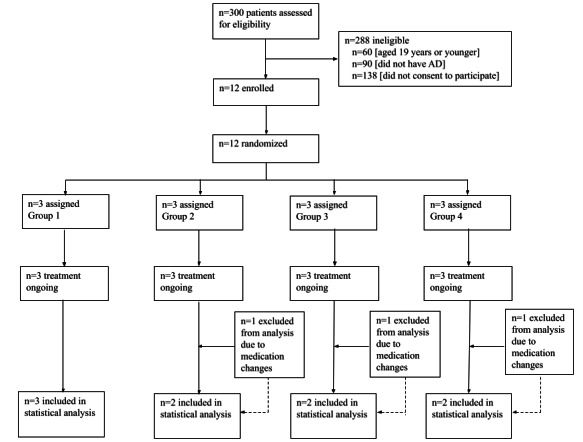
Trial profile. AD: atopic dermatitis.

A total of 12 adults (mean age 31.1 years; range 20-43 years) were recruited between August and November 2022 (Figure 3). Of the 12 participants, 2 (17%) had mild AD, 7 (58%) had moderate AD, and 3 (25%) had severe AD. More details regarding the sample characteristics are presented in Table 1.

**Table 1:** Sample characteristics of the participants (N=12).

Characteristics			Frequency, n (%)
**Sex**			
	Female	5 (42)	
	Male	7 (58)	
**Age (years)**			
	20-29	5 (42)	
	30-39	5 (42)	
	40-49	2 (17)	
**Education level**			
	High school graduate or less	4 (33)	
	Currently enrolled in or graduated from college	7 (58)	
	Currently enrolled in or graduated from graduate school	1 (8)	
**Severity of atopic dermatitis**			
	Mild	2 (17)	
	Moderate	7 (58)	
	Severe	3 (25)	
**Duration of disease (years)**			
	≤10	1 (8)	
	11-20	4 (33)	
	21-30	6 (50)	
	>30	1 (8)	
**Comorbidity of other allergic diseases**			
	Atopic dermatitis only	3 (25)	
	Comorbid with other allergic diseases	9 (75)	
**Family history of allergic diseases**			
	Atopic dermatitis	4 (33)	
	Allergic rhinitis	4 (33)	
	Food allergy	1 (8)	
	None	3 (25)	
**Alcohol consumption frequency over the past year**			
	Not at all in the past year	4 (33)	
	Less than once a month	1 (8)	
	About once a month	1 (8)	
	2-4 times a month	2 (17)	
	2-3 times a week	4 (33)	
**Health literacy**			
	Health literacy (score 15 out of 15)	9 (75)	

### Primary Outcome

**Figure 4:**
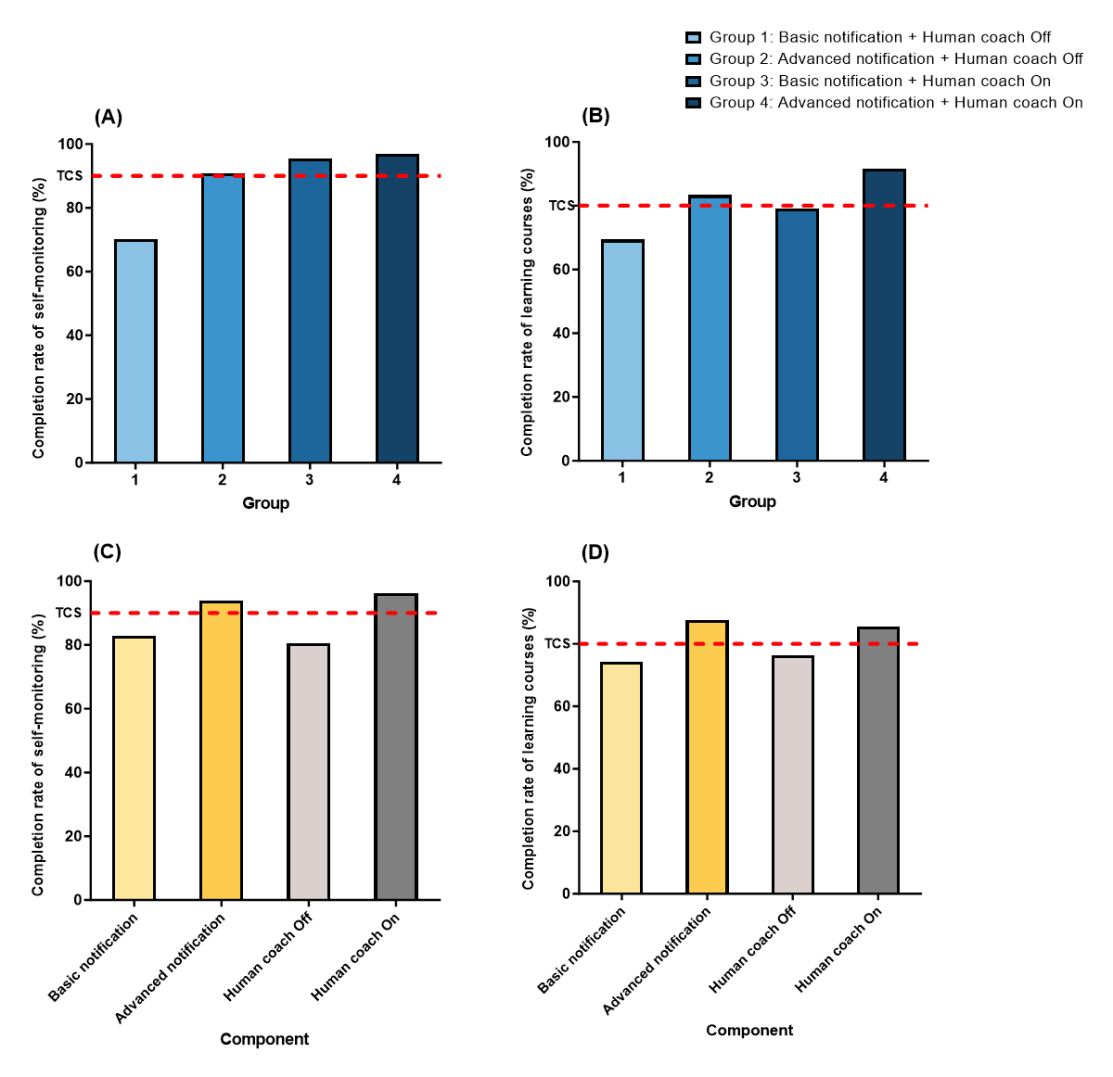
Group-specific (A) and (B) and component-specific (C) and (D) user engagement, measured by in-app behavioral data on core intervention components (ie, completion rate of self-monitoring and learning courses) after the 8-week intervention period.

Regarding the user engagement rates among different groups (Figures 4A and 4B), groups 2 (90.9%), 3 (95.5%), and 4 (97%) showed higher completion rates for self-monitoring compared to the predetermined TCS (90%). Additionally, groups 2 (83.3%) and 4 (91.7%) had higher completion rates for learning courses than the TCS (80%). These results indicate that group 4, provided with advanced-level push notifications and a human coach, had the highest user engagement during the intervention.

As shown in Figures 4C and 4D, “advanced-level push notification” (93.9%) and “human coach on” (96.2%) were the experimental intervention components that exceeded the predetermined TCS for self-monitoring (90%). The experimental intervention components that exceeded the TCS for learning courses (80%) were also “advanced-level push notification” (87.5%) and “human coach on” (85.4%). Overall, “advanced-level push notification” and “human coach on” demonstrated the highest user engagement among the experimental intervention components.

### Secondary Outcome

Qualitative data were organized into three key themes: (1) perceived acceptability of the experimental intervention components, (2) satisfaction of the core intervention components, and (3) suggestions for improvement in the overall intervention program. Table 2 presents all themes and subthemes with corresponding quotes.

**Table 2:** Key themes, subthemes, and quotes from semistructured interviews.

Themes, subthemes, and components					Verbatim examples		
**Key theme 1: perceived acceptability of the experimental intervention components**							
	**Push notification component**						
		**Technical aspect**					
			Basic push notification	It would be better if we could choose the time to receive notifications, and it would be better if we could receive the notification functioned similarly to a wake-up alarm that rings again if not checked...			
			Advanced push notification	I lead a busy life, so receiving notifications was helpful. In fact, I think it was better for me to receive notifications frequently.			
		**Content aspect**					
			Basic push notification	The (content) of the notifications was all good. It would be nice to have additional features like setting reminders for taking medication. Or maybe some information on whether I've applied moisturizer or taken my medicine today. Something like that would be useful.			
			Advanced push notification	The notification content was good enough as it was, with just simple and neat notifications.			
	**Human coach component**						
		**Technical aspect**					
			Human coach off	It would be better if there was a feature that allowed patients to send messages to report any technical errors or issues...It might be better to communicate using this feature.			
			Human coach on	I wish there was a channel where atopic patients could communicate with each other.			
		**Content aspect**					
			Human coach off	It would be great if we could receive feedback for emergency situations.			
			Human coach on	For example, it would be more effective to ask direct questions like 'have you reduced your medication dosage?' rather than asking about difficulties or inconveniences...			
**Key theme 2: satisfaction with the core intervention components**							
	**Self-monitoring**						
		Building health habits		I used to forget to take my medicine, but ever since I started using the app to check it, I've been taking it every morning and before bed, and I've been doing it consistently. I've established a routine of recording it separately from the app. By recording the questionnaire every day, I can now monitor the daily improvement or worsening of my condition, which was the best part of the app.			
	**Learning course**						
		Acquiring reliable information		It was great to learn about the parts that I used to miss with reliable information.			
**Key theme 3: suggestions for improvement on the overall intervention program**							
	Diversity of daily self-monitoring form questions				I wish, that depending on the symptoms, different questions would be asked to determine whether the symptoms improved or worsened from the previous day.		
	Burdensomeness of self-monitoring feature				Taking pictures of my body to check the skin lesion was burdensome. The questionnaire was too lengthy.		
	Not tailored contents				There was information that would have been useful, if symptoms hadn't been so severe. It would be better to recommend it to patients with mild symptoms. The quiz following the video was so simple that I didn't even need to watch it and could simply answer the questions correctly. This is why I stopped watching the weekly videos.		
	Motivating factors				It would be great to include elements that can boost motivation, such as fun factors or any benefits.		
	Technical issues				There were times when I couldn't continue with the survey for a few days because some questionnaire items wouldn't move forward at all. So, it would be great if those issues could be improved.		

#### Key Theme 1: Perceived Acceptability of the Experimental Intervention Components

Perceived acceptability was measured using the components’ technical and content aspects. Regarding the technical aspect of the push notification component, 60% (3/5) participants receiving “basic-level push notifications” responded that they would like the push notification frequency to increase. Moreover, 20% (1/5) responded that it would be better to select the time and frequency of the push notifications and be reminded if they did not complete the task. And 50% (2/4) receiving “advanced-level push notifications” were overall satisfied with the current push notification frequency. Regarding the content aspect of this component, both groups responded that they were satisfied with the provided notification contents. However, 20% (1/5) of participants in the group receiving “basic-level push notifications” suggested that it would be helpful to receive a push notification reminding them to take medication or apply some moisturizer.

Regarding the technical aspect of the human coach component, 40% (2/5) participants assigned to the “human coach off” component requested a 1:1 communication channel within the app, as they could not receive assistance from a human coach. A total of 75% (3/4) of participants assigned to the “human coach on” component preferred to have an in-app communication channel rather than using a different instant messaging app (ie, KakaoTalk) for communication with a human coach. And 25% (1/4) of participants also suggested integrating a community feature for patients to communicate with each other. Regarding the content aspect of the human coach component, 20% (1/5) of participants assigned to the “human coach off” suggested adding a telehealth feature for emergencies. And, 25% (1/4) participants assigned to the “human coach on” preferred the coach asking specific questions related to symptom management, such as “Have you taken your medicine today?” or “Have you visited the hospital?” rather than the questions relevant to the app use, like “Is there anything difficult or uncomfortable while using the app?”

#### Key Theme 2: Satisfaction With the Core Intervention Components

Satisfaction was measured for each core intervention component, self-monitoring, and learning courses. Regarding the self-monitoring component, 78% (7/9) of participants reported that self-monitoring helped build health habits, including better medication adherence, reduced scratching behavior, and consistent use of moisturizers. Moreover, they could easily track their symptoms through weekly reports, which helped them monitor their symptoms over time. Regarding the learning course component, 56% (5/9) of participants indicated that they acquired reliable information through weekly videos.

#### Key Theme 3: Suggestions for Improvement on Overall Intervention Program

Suggestions for improvement in the overall intervention program were divided into 5 subthemes. First of all, 33% (3/9) of participants recommended diversifying the questions of the daily self-monitoring form, as they found them to be repetitive and lacking in variation. Second, 22% (2/9) of participants found the self-monitoring burdensome as they had to upload lesion pictures daily. Third, 44% (4/9) of participants felt the learning course was not sufficiently tailored to their needs. They found the video content insufficiently helpful for patients with severe disease; the postquiz questions were unchallenging; and the video was too long. Fourth, 11% (1/9) of participants suggested adding motivating factors to the intervention program to make them more engaged with the app. Lastly, technical issues within the app were mentioned. A total of 33% (3/9) of participants recommended improving its performance, such as fixing bugs in the self-monitoring feature, reducing duplicate push notifications, and improving video sound quality.

### Clinical Outcomes

Descriptive statistics, for example, mean (SD), were used to analyze clinical outcomes by group and experimental intervention component. The ISI score showed the greatest improvement in group 2 (mean change –4.50, SD 6.36). The EASI, POEM, and BFNE scores showed the highest improvement in group 3 (mean change –10.20, SD 9.90, mean change –3.00 score, SD 15.56, and mean change –4.50, SD 6.36, respectively). The PSS and DLQI scores presented the greatest improvement in group 4 (mean change –3.50, SD 3.54, and mean change –6.00, SD 11.32, respectively).

**Figure 5:**
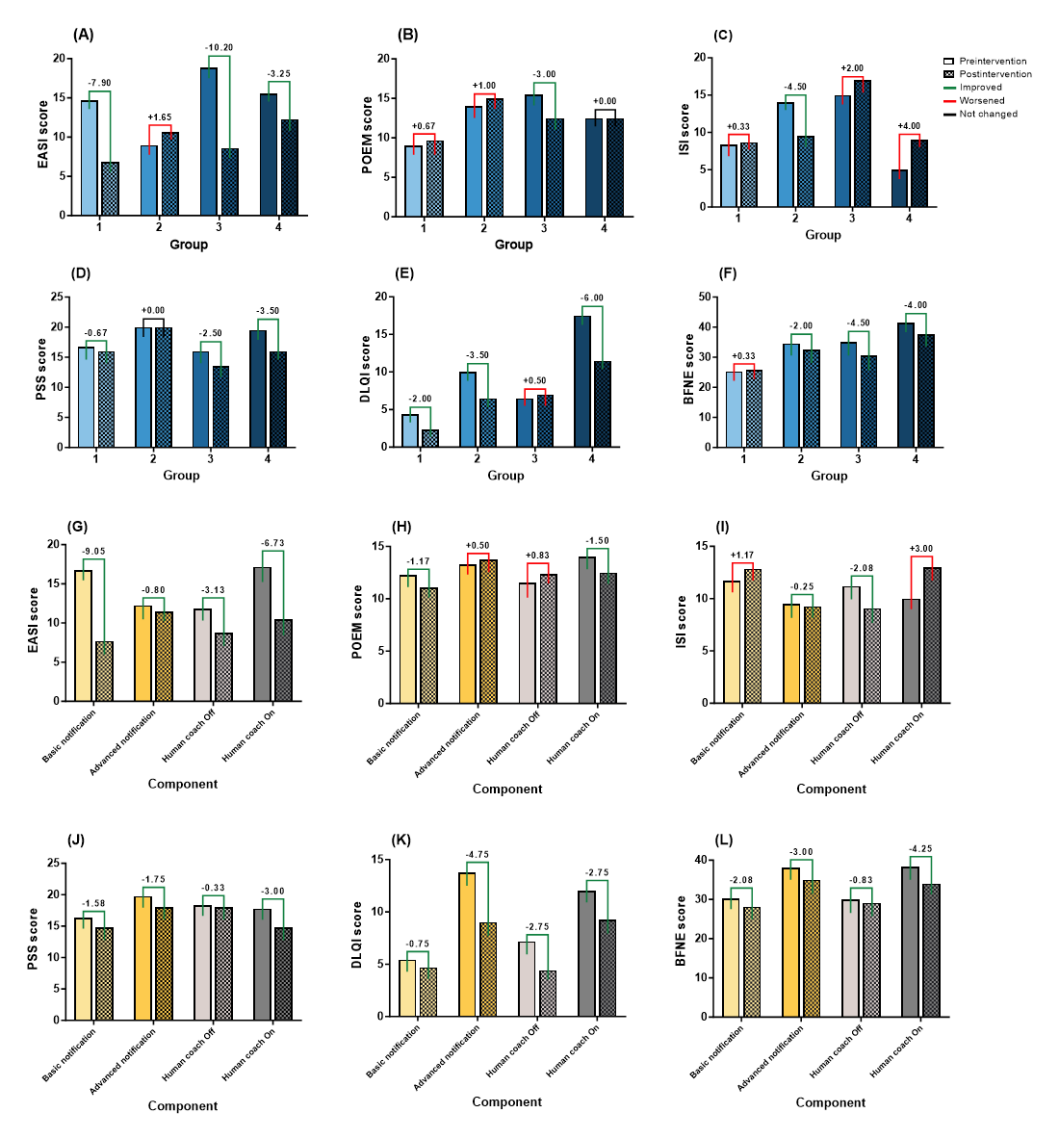
Differences in clinical outcomes between groups (A-F) and components (G-L) from preintervention to postintervention at 8 weeks. BFNE: brief fear of negative evaluation; DLQI: dermatology life quality index; EASI: eczema area and severity index; ISI: insomnia severity index; POEM: patient-oriented eczema measure; PSS: perceived stress scale.

Regarding the push notification component, the ISI, PSS, DLQI, and BFNE scores showed the highest improvement in the “advanced-level push notification” component (mean change –0.25, SD 6.34, mean change –1.75, SD 2.99, mean change –4.75, SD 8.06, and mean change –3.00, SD 3.46, respectively). Regarding the human coach component, the EASI, POEM, PSS, and BFNE scores presented the highest improvement in the “human coach on” component (mean change –6.73, SD 7.41, mean change –1.50, SD 11.73, mean change –3.00, SD 2.94, and mean change –4.25, SD 3.69, respectively). More detailed results can be found in Figure 5.

## Discussion

### Principal Findings

Our primary objective was to investigate how the perceived acceptability of experimental intervention components and satisfaction with core intervention components affect user engagement in DTx. We examined in-app behavioral data on core intervention components (ie, percentages of self-monitoring forms and learning courses completed) as a user engagement metric. As hypothesized, the TCS of user engagement was achieved in group 4, where all 2 experimental factors were advanced simultaneously. Furthermore, clinical outcomes related to the mental health of patients with AD improved in group 4. This study identified potential barriers and facilitators of user engagement through semistructured interviews on the patients’ satisfaction with core intervention components. Overall, our analysis of Atomind data suggests that incorporating advanced-level push notifications with a human coach, tailoring contents with various self-monitoring tools, and implementing some motivational factors (eg, rewards) may improve user engagement.

### Comparison With Previous Work

To the best of our knowledge, this is the first study to examine the impact of different levels of push notification, human coach, and satisfaction with core intervention components on user engagement in DTx using mixed methods. Although there is a proliferation of clinical research on user engagement with mobile health apps, the majority only conducted traditional RCTs [28-39] or optimization trials with a single type of assessment method [40-44]. The findings from these earlier studies with traditional RCTs only explained how the intervention as a package affected user engagement; they could not identify the specific intervention elements that impacted it [29]. Additionally, only assessing quantitative data from optimization trials (eg, factorial experiments) limits the understanding of barriers and facilitators affecting user engagement [45,46]. In contrast, this study clearly showed that advanced-level push notifications and communication with a human coach are the main factors enhancing user engagement. Furthermore, our qualitative analysis showed that advanced-level push notifications were sufficient in frequency to serve as a reminder in busy daily lives, and their content was concise enough to be acceptable. Although communication with a human coach improved user engagement, our qualitative findings suggest that the human coach platform should have been implemented in the internal system of the Atomind app with more diverse questions and detailed responses. Using a mixed methods approach to assess various factors contributing to user engagement in Atomind enabled us to gain insights into the “what, how, and why” of this phenomenon, which is critical to figuring out what steps must be taken to improve an intervention.

Establishing a TCS for user engagement has been applied, as this study is a proof-of-concept study with a small sample size. This approach allows for resource-efficient research with clear go-or-no-go decision-making, lowering the risk of confirmatory bias [19]. Concerning TCS determination, each previous study had its own logic established and multiple metrics to account for user engagement [33,47]. This is because user engagement is a multifaceted concept with no universal consensus on how to perceive it [6,7,34]. Among the various metrics of user engagement from previous research, the completion of specific activities or modules of the intervention was the most commonly used metric for user engagement [29-34,36,39,43,48]. Similarly, we measured user engagement in the app by assessing the completion rate of the core intervention components. In this study, self-monitoring is for daily activity, while learning courses are for weekly activity. Thus, we set up different levels of TCS for each activity to assess user engagement; the completion rate was 90% for self-monitoring forms and 80% for learning courses.

Regarding the clinical outcomes from this study, people who received the advanced level of experimental intervention components saw improvement in the majority of psychological symptoms (eg, stress, quality of life, and fear of negative evaluation), which was more than the physical symptoms related to AD. These findings correspond with previous research that suggests digital interventions should focus mainly on improving mental health conditions to support better physical health conditions [49,50]. This trend is caused by several inherent factors of mental health interventions, including the stigma associated with mental problems and diagnosis-specific barriers to accessing mental health services [51]. Likewise, Atomind is a digital intervention for patients with AD that encourages healthy behaviors and mental health conditions for effective symptom management. Thus, improving psychological measures by engaging with Atomind indicates that it achieved the intended proximal outcome.

### Limitations and Future Directions

First, the statistical power of this study is insufficient to determine significant effects before and after the intervention. However, setting reasonable TCS for quantitative data and collecting qualitative data will support our findings on DTx optimization for use in well-powered RCTs. Second, the Atomind app is only available for use on the Android operating system. To overcome this limitation, we provided Android smartphones during the intervention period to those (n=5) who had other operating systems on their smartphones. Despite this effort, the user experience with Atomind, which is closely related to user engagement, may be affected. Lastly, technical issues with the app occurred frequently during the intervention period, which may affect user engagement. As Atomind was in the development phase, these problems could have taken place; however, its technical system should be improved in a later version and used for future clinical research.

### Conclusions

This proof-of-concept, mixed methods study with an experimental 2 × 2 factorial design demonstrates the impact that perceived acceptability of experimental intervention components and satisfaction with core intervention components in DTx have on user engagement. The findings will be used to refine the intervention and inform the design of the next RCT to test its effectiveness. Furthermore, this research design may serve as a model for how to examine and optimize overall engagement in DTx in broad terms; it will help future research investigate the complex relationship between engagement and clinical outcomes.

## Appendix

Multimedia Appendix 1 Overview of push notifications between two groups.

Multimedia Appendix 2 Atomind app sample screen.

Multimedia Appendix 3 Overall topics of weekly videos.

Multimedia Appendix 4 Semi-structured interview guideline.

Multimedia Appendix 5 Assessment methods and assessment period for each measurement.

## Abbreviations

AD: atopic dermatitis
ADHD: attention-deficit/hyperactivity disorder
BFNE: brief fear of negative evaluation
CBT: cognitive behavioral theory
DLQI: Dermatology Life Quality Index
DTx: digital therapeutics
EASI: eczema area and severity index
ISI: insomnia severity index
MOST: multiphase optimization strategy
POEM: patient-oriented eczema measure
PSS: perceived stress scale
RCT: randomized controlled trial
TCS: threshold of clinical significance

## Data Availability

The data sets generated and/or analyzed during this study are not publicly available due to the need to maintain privacy and confidentiality, but are available from the corresponding author on reasonable request. Requests for access to specific data points or additional information will be considered on a case-by-case basis.
