# Supplementary material for: The Impact of Intervention Design on User Engagement in Digital Therapeutics Research: Factorial Experiment With a Mixed Methods Study
Source: JMIR Form Res. 2024 Feb 9;8:e51225. doi: 10.2196/51225 (PMC10891489; doi:10.2196/51225)
Supplement: Multimedia Appendix 1 [file formative_v8i1e51225_app1.docx]

**Appendix 1. Overview of push notifications between two groups**

| **Category** | | **Push Notification Type** | **Setting** | **Group** | |
| --- | --- | --- | --- | --- | --- |
|  |  |  |  | **Basic level** | **Advanced level** |
| Self-monitoring | Baseline assessment | Baseline assessment notification | 8:30 AM (default / editable) | + | + |
|  |  |  |  |  |  |
|  |  | Reminder to encourage users to complete a baseline assessment (given if users stop completing the baseline assessment) | 8:30 AM (default / editable) | + | + |
|  |  |  |  |  |  |
|  | Self-monitoring form (daily/weekly/monthly) | Self-monitoring notification | 8:30 AM (default / editable) | + | + |
|  |  |  |  |  |  |
|  |  | Reminder (given if users do not complete the previous day's assessment) | 8:30 AM (default / editable) | - | + |
|  |  | Reminder to encourage users to complete the self-monitoring form | 8:30 PM (fixed) | - | + |
|  |  |  |  |  |  |
| Personalized feedback report | | Report issued | when report is issued | + | + |
|  |  |  |  |  |  |
| Learning course | | Weekly class notification | Mon/Wed 6:00 PM  (default / editable) | + | + |
|  |  |  |  |  |  |
|  |  | Deadline for the class approaching (given if users do not finish the weekly class) | Day 5, 6, and 7 every week (Time: identical to Weekly class notification) | - | + |
|  |  |  |  |  |  |
|  |  | Deadline for the class approaching (given if users do not log in within two hours after receiving the weekly class notification) | Day 5, 6, and 7 every week  (Time: Weekly class notification + 2 hour) | - | + |
| Mission | | Mission Notification | 12:00 PM (fixed)  (given once the day after the start of the learning course) | - | + |
|  |  |  |  |  |  |
|  |  | Reminder (given if users do not complete the mission) | Day 5, 6, and 7 every week 12:00 PM | - | + |
|  |  |  |  |  |  |
|  |  | Reminder to encourage users to complete the mission | Day 5, 6, and 7  every week 12:00 PM (if users do not log in after receiving the notification, an additional notification is given at 2:00 PM) | - | + |
|  |  | Mission: behavioral experiment  (recording results after the mission) | 24 hours after completing the mission | + | + |
|  |  |  |  |  |  |
|  |  | Mission: behavioral experiment  (if users do not record results) | One day before the mission deadline | - | + |

*+ implies *given* and - implies *not given*
