# Supplementary figures and images for: The Impact of Intervention Design on User Engagement in Digital Therapeutics Research: Factorial Experiment With a Mixed Methods Study
Source: JMIR Form Res. 2024 Feb 9;8:e51225. doi: 10.2196/51225 (PMC10891489; doi:10.2196/51225)

**Appendix 2.** ***Atomind* app sample screen**

**
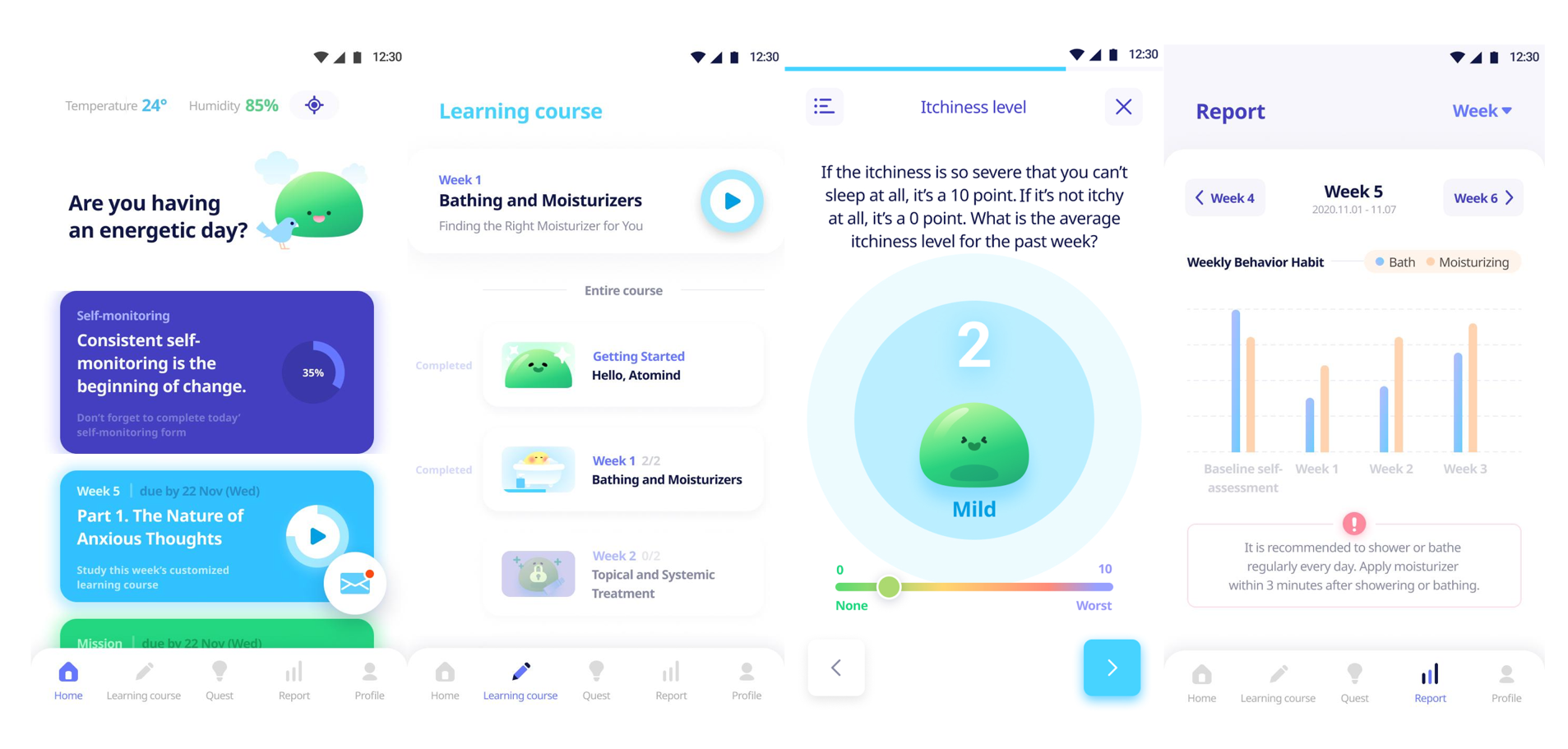
**

Supplement: Multimedia Appendix 2 [file formative_v8i1e51225_app2.docx]
