# Supplementary material for: The Impact of Intervention Design on User Engagement in Digital Therapeutics Research: Factorial Experiment With a Mixed Methods Study
Source: JMIR Form Res. 2024 Feb 9;8:e51225. doi: 10.2196/51225 (PMC10891489; doi:10.2196/51225)
Supplement: Multimedia Appendix 3 [file formative_v8i1e51225_app3.docx]

**Appendix 3. Overall topics of weekly videos**

| **Curriculum** | | | |
| --- | --- | --- | --- |
| **Week** | **Category** | **Topics** | **Mission** |
| 1 | Introduction | App introduction and instruction for use | X^3^ |
|  | Dermatology | Bathing and Moisturizing | X |
| 2 |  | Topical treatment and Systemic treatment | X |
| 3 |  | Food and Environment | X |
| 4 | Psychiatry | Cognitive-Behavioral Therapy for insomnia/depression/anxiety/itching^1^ | O |
| 5 |  |  |  |
| 6 |  | Mindfulness: Introduction and Raisin Meditation | O |
| 7 |  | Mindfulness: Sitting meditation, Habit reversal training (HRT) | O |
| 8 | Finishing | Wrap-up session | X |
|  | Psychiatry^2^ | Cognitive-Behavioral Therapy for insomnia/depression/anxiety/itching | O |
|  |  | Mindfulness: Noticing Body sensations, Breathing relaxation techniques | O |
|  |  | Mindfulness: Sensory Walk and Wrap-up | O |

^1^Among four sessions, two sessions are chosen based on the result of users' baseline assessment
^2^Week 8 Psychiatry sessions are not mandatory
^3^O implies *included* and X implies *not included*
