# Supplementary material for: The Impact of Intervention Design on User Engagement in Digital Therapeutics Research: Factorial Experiment With a Mixed Methods Study
Source: JMIR Form Res. 2024 Feb 9;8:e51225. doi: 10.2196/51225 (PMC10891489; doi:10.2196/51225)
Supplement: Multimedia Appendix 4 [file formative_v8i1e51225_app4.docx]

**Appendix 4.** **Semi-structured interview guideline**

Hello, OOO.

Thank you for taking the time to participate in this interview and provide feedback on *Atomind*, the application for managing atopic dermatitis. I am OOO, and I will be conducting this in-depth interview with you.

The purpose of this interview is to collect feedback from users like you and identify the effectiveness of the intervention program, as well as any suggestions for improvement.

Specifically, we would like to hear about any changes in symptom management that you have experienced after using the *Atomind* app for the past eight weeks.

To ensure that we do not miss any valuable insights from you today, this interview will be recorded. However, the final document will not contain any identifiable information and will be kept strictly confidential. The document will be managed by the *Atomind* research team at Yonsei University, and used to improve the *Atomind* program in the future. This interview is expected to last approximately 20 minutes, and we will provide compensation after the interview.

#### ***Warm-up questions***

1. Which aspects of symptom management have changed the most after eight weeks of treatment compared to the past? *(For example, changes in bathing methods, medication usage, stress management techniques, etc.)*
2. What specific efforts have you made to achieve the changes you mentioned earlier in managing your symptoms? *(For example, how have you changed your bathing routine, how often do you take prescribed medications, or what new stress management techniques have you implemented, etc.)*
   1. What has been the most difficult obstacle for you as you continue to make these efforts? *(For example, weather conditions, a busy daily schedule, concerns about people around you, etc.)*

#### ***Section 1: Acceptability of experimental intervention components (i.e., push notification and human coach)***

1. How helpful was the **push notification component** for reminding you to complete the given tasks for your symptom management?
2. How helpful was the **human coach component** (*Human coach on* group: using KakaoTalk) for your symptom management?

#### ***Section 2: Satisfaction of core intervention components (i.e., self-monitoring and learning course)***

1. How satisfied were you with the **self-monitoring** feature in bringing about the changes in your symptom management?
2. How satisfied were you with the **learning course** feature in bringing about the changes in your symptom management?
3. Besides the core intervention components (i.e., in-app self-monitoring, learning courses), were there any other intervention components that were particularly satisfied in managing your symptoms?

#### ***Section 3: Suggestions of overall intervention program***

1. Do you have any suggestions for improving the *Atomind* app to manage your symptoms effectively?

Thank you sincerely for participating in this interview. We will request several documents from you for compensation.
