# Supplementary material for: The Impact of Intervention Design on User Engagement in Digital Therapeutics Research: Factorial Experiment With a Mixed Methods Study
Source: JMIR Form Res. 2024 Feb 9;8:e51225. doi: 10.2196/51225 (PMC10891489; doi:10.2196/51225)
Supplement: Multimedia Appendix 5 [file formative_v8i1e51225_app5.docx]

**Appendix 5. Assessment methods and assessment period for each measurement**

| **Outcome measure** | **Assessment method** | **Assessment administrator** | **Assessment Period** | | |
| --- | --- | --- | --- | --- | --- |
|  |  |  | **baseline** | **4 weeks** | **8 weeks** |
| **Primary Outcome** | | | | | |
| Acceptability, usability, and satisfaction | Semi-structured interview | in person by researchers |  |  | x |
| **Secondary outcome** | | | | | |
| Severity of Atopic dermatitis | Eczema Area and Severity Index (EASI) | in person by dermatologists | x | x | x |
| Atopic eczema severity reported by patients | Patient-Oriented Eczema Measure (POEM) | in-app weekly assessment by self-report | x | x | x |
| Insomnia severity | Insomnia Severity index (ISI) | in-app monthly assessment by self-report | x | x | x |
| Perceived stress | Perceived Stress Scale (PSS) | in-app monthly assessment by self-report | x | x | x |
| Quality of life | Dermatology Life Quality Index (DLQI) | in paper by self-report | x | x | x |
| Fear of negative evaluation | Brief Fear of Negative Evaluation Scale (BFNE) | in paper by self-report | x | x | x |
